# Searching for HIV and AIDS Health Information in South Africa, 2004-2019: Analysis of Google and Wikipedia Search Trends

**DOI:** 10.2196/29819

**Published:** 2022-03-11

**Authors:** Babatunde Okunoye, Shaoyang Ning, Dariusz Jemielniak

**Affiliations:** 1 Berkman Klein Centre for Internet and Society Harvard University Cambridge, MA United States; 2 Department of Journalism, Film and Television University of Johannesburg Johannesburg South Africa; 3 Department of Mathematics and Statistics Williams College Massachusetts, MA United States; 4 Management in Networked and Digital Societies Department Kozminski University Warsaw Poland

**Keywords:** HIV/AIDS, web search, big data, public health, Wikipedia, information seeking behavior, online behavior, online health information, Google Trends

## Abstract

**Background:**

AIDS, caused by HIV, is a leading cause of mortality in Africa. HIV/AIDS is among the greatest public health challenges confronting health authorities, with South Africa having the greatest prevalence of the disease in the world. There is little research into how Africans meet their health information needs on HIV/AIDS online, and this research gap impacts programming and educational responses to the HIV/AIDS pandemic.

**Objective:**

This paper reports on how, in general, interest in the search terms “HIV” and “AIDS” mirrors the increase in people living with HIV and the decline in AIDS cases in South Africa.

**Methods:**

Data on search trends for HIV and AIDS for South Africa were found using the search terms “HIV” and “AIDS” (categories: health, web search) on Google Trends. This was compared with data on estimated adults and children living with HIV, and AIDS-related deaths in South Africa, from the Joint United Nations Programme on HIV/AIDS, and also with search interest in the topics “HIV” and “AIDS” on Wikipedia Afrikaans, the most developed local language Wikipedia service in South Africa. Nonparametric statistical tests were conducted to support the trends and associations identified in the data.

**Results:**

Google Trends shows a statistically significant decline (*P*<.001) in search interest for AIDS relative to HIV in South Africa. This trend mirrors progress on the ground in South Africa and is significantly associated (*P*<.001) with a decline in AIDS-related deaths and people living longer with HIV. This trend was also replicated on Wikipedia Afrikaans, where there was a greater interest in HIV than AIDS.

**Conclusions:**

This statistically significant (*P*<.001) association between interest in the search terms “HIV” and “AIDS” in South Africa (2004-2019) and the number of people living with HIV and AIDS in the country (2004-2019) might be an indicator that multilateral efforts at combating HIV/AIDS—particularly through awareness raising and behavioral interventions in South Africa—are bearing fruit, and this is not only evident on the ground, but is also reflected in the online information seeking on the HIV/AIDS pandemic. We acknowledge the limitation that in studying the association between Google search interests on HIV/AIDS and cases/deaths, causal relationships should not be drawn due to the limitations of the data.

## Introduction

A major obstacle to combating the impacts of disease in developing countries is the paucity of high-quality health data, including data regarding people’s health information needs [1]. If they do not understand people’s everyday concerns, health organizations and policy makers are less able to effectively target education and programming efforts for all genders and age groups [1]. People’s information needs and their everyday concerns are often expressed via search engine queries as millions go online to meet their health information needs.

HIV/AIDS remains a major global public health concern, despite concerted efforts aimed at combating the disease. There are an estimated 37.9 (32.7-44.0) million people worldwide living with HIV, the virus that causes AIDS [2]. Sub-Saharan Africa has the greatest burden of HIV/AIDS, with South Africa bearing the greatest burden of the disease in the world. An estimated 7.7 million people were living with HIV and AIDS in South Africa in 2018, with an adult prevalence rate of 20.4% [2]. In 2018, approximately 71,000 South Africans died of AIDS-related causes.

Considerable progress has been made in the fight against HIV/AIDS globally. Globally, an estimated 37.9 (32.7-44.0) million people were living with HIV in 2018 [2], an increase from previous years (eg, 35.3 million in 2012), as more people receive life-saving antiretroviral therapy. At the same time, the number of AIDS-related deaths is also declining, with 770,000 deaths in 2018 [2], down from 1.6 (1.4-1.9) million deaths in 2012 and 2.3 (2.1-2.6) million in 2005 [3]. Gains have also been made toward many of the 2020 Sustainable Development Goals targets and elimination commitments, although significant challenges remain [4]. This is within the context of high-risk behavior among people living with HIV in low- and middle-income countries [5] and insufficient human resources in HIV care in low- and middle-income countries [6].

A key component of the fight against HIV/AIDS has been behavioral interventions [3]. A global meta-analysis of studies determined that behavioral interventions reduce sexual risk behavior and reduce sexually transmitted infections and HIV [3]. Behavioral interventions typically involve harmonizing messages and the dissemination of information about HIV transmission and various prevention approaches using television, radio, outdoor advertising, and information and communications technology. The outcome of all these behavioral interventions is that in sub-Saharan Africa, the percentage of young people (15-24 years) demonstrating a comprehensive and accurate understanding of HIV has generally increased over the years. For example, this understanding rose by 5 percentage points for men and by 3 for women from 2002 to 2011, although knowledge levels remain low (36% for young men and 28% for young women) [3].

In South Africa, there have been a number of nationwide communication campaigns related to raising awareness of HIV and AIDS. Soul City and MTV Shuga are two examples of such multimedia campaigns that promote good sexual health and well-being and have effective outcomes [7]. Research into the impact of the Soul City and other communication campaigns found that they had a positive effect on the sexual behavior of adults that had been exposed to the campaign message, for instance by bringing about positive changes in condom use and HIV testing [8].

The combined effects of these awareness campaigns have been an increase in knowledge and understanding of HIV/AIDS. One likely outcome of an increase in awareness of HIV/AIDS in South Africa is that a percentage of the population will turn to the internet via search engines for information on HIV/AIDS. Internet penetration in South Africa is 56% (32.9 million) [9].

An increasing number of people are using the internet to support their health care needs [10,11] and search engines such as Google, Yahoo! and Bing are often their first port of call. One of the early studies on search engine use found that 1 in every 28 (3.5%) pages viewed on the web is a search results page, making the use of a search engine the second most popular internet task next to email [12]. Another early study found that there are over 6.75 million health-related internet searches carried out every day worldwide, representing approximately 5% of all internet searches [13]. Search engine use is the most common approach to online information seeking [14], and a study by the Pew Research Center found that half of all internet users now use search engines on a typical day [15]. It has also been estimated that about 1 billion Google searches daily are health-related, which is about 7% of all daily searches [16].

This paper examines trends in online search on HIV and AIDS on Google, which is the dominant search engine worldwide [17] and is the top search engine in most countries with a few exceptions, such as Russia (Yandex) and China (Baidu). In South Africa, Google is also the dominant search engine [17]. Google search data have been used for understanding health information needs, health surveillance, and health forecasting [10,18-23]. Although the use of Google search engine data for health and development in general has been criticized [24,25] in recent years due to an unbalanced and opaque approach that did not satisfy scientific transparency, in recent years, organizations such as the World Bank [26,27] and several government departments around the world have begun revisiting the behavioral big data methods of search engine data in aid of international development. Drawing on feedback from the scientific community on the best practices of big data research [24,25,28,29], particularly paying attention to integrating both big data and “small data” (eg, survey data and other traditional data collection mechanisms) to create more complete and accurate data sets, they have made significant advances in this field. This paper advances the hypothesis that the online search behavior of people looking for health information can be used to understand their health information needs, and also for health surveillance and monitoring [10,18-23].

## Methods

The methods employed in this study were derived from the considerable literature regarding using search data to understand the health information needs of the public and for health surveillance [10,18-23]. The first step involved search term selection. The search terms chosen as proxies to gauge public interest in HIV and AIDS in South Africa were “HIV” and “AIDS.” These were the only search terms selected given the specificity of the topics concerned. Data [30] on online searches for the terms “HIV” and “AIDS” were thus obtained on Google Trends (categories: health, web search) to visualize how searches were conducted on both search terms. The data were obtained for the years 2004-2019. Some of the most important search queries (top and rising) searched for by South Africans on “HIV” and “AIDS” were downloaded from the Google Trends data set [30] and recorded. Data on regional interest in both search terms for South Africa were downloaded. In addition, the top and rising search queries related to “HIV” and “AIDS” were also accessed via Google Trends.

The nonparametric Mann-Kendall test [31] was conducted to evaluate the significance of trends. The Mann-Kendall test accounts for the autocorrelation of time series (for the alternative hypothesis of monotonic trends) by the design of its test statistic on the time series increments. The Mann-Kendall test was adapted based on the log ratio of search volumes between search terms to evaluate their associations (between “HIV” and “AIDS”) and to investigate the search trend of the term “AIDS” with adjustment (log ratio between “flu” and “AIDS”).

Nonparametric Mann-Kendall tests were conducted to indicate the significant decline in AIDS-related deaths in South Africa and the increasing trend for the number of estimated adults and children living with HIV. Permutation test based on nonparametric Spearman rho [32] was conducted to verify the association between Google search interest for the term “AIDS” and AIDS-related deaths in Africa. Due to the difference in time frequency, monthly Google search data for the term “AIDS” were aggregated through moving average to estimate yearly search interest. All statistical analysis was conducted with R (version 4.1.2; R Foundation for Statistical Computing); Mann-Kendall tests were done using the *trend* package and the Spearman test was performed using the *stats* package.

Pageviews for the topics “HIV” and “AIDS” from 2015-2020 in Wikipedia Afrikaans, the most developed indigenous language Wikipedia service in South Africa, were obtained from Wikipedia [33]. The year with the earliest data on HIV and AIDS on Wikipedia Afrikaans is 2015.

Review studies on the use of search engine data to understand public interests and trends have noted the “traps in big data” [24,25,28,29], emphasizing that data sources such as search engines are best used to supplement traditional data sources like surveys and that, in general, confidence in such data is strengthened when their findings can be replicated using other data sources and platforms. Thus, this paper also obtained and plotted data [34] on the estimated number of adults and children living with HIV, as well as AIDS-related deaths in South Africa, from the United Nations Joint Programme on HIV/AIDS for the years 2004-2019.

## Results

**Figure 1:**
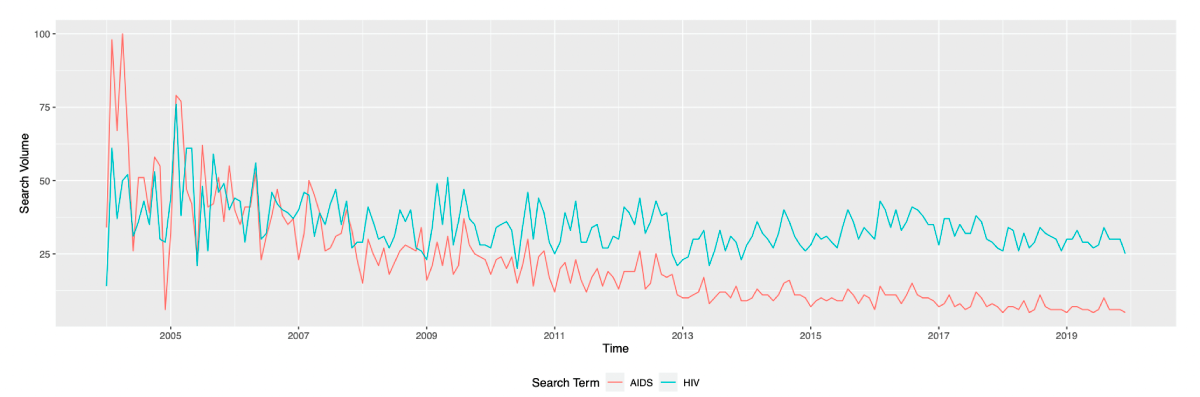
Search interest in the search terms "HIV" and "AIDS" (category: health, web search) in South Africa, 2004-2019 (accessed December 24, 2020).

**Figure 2:**
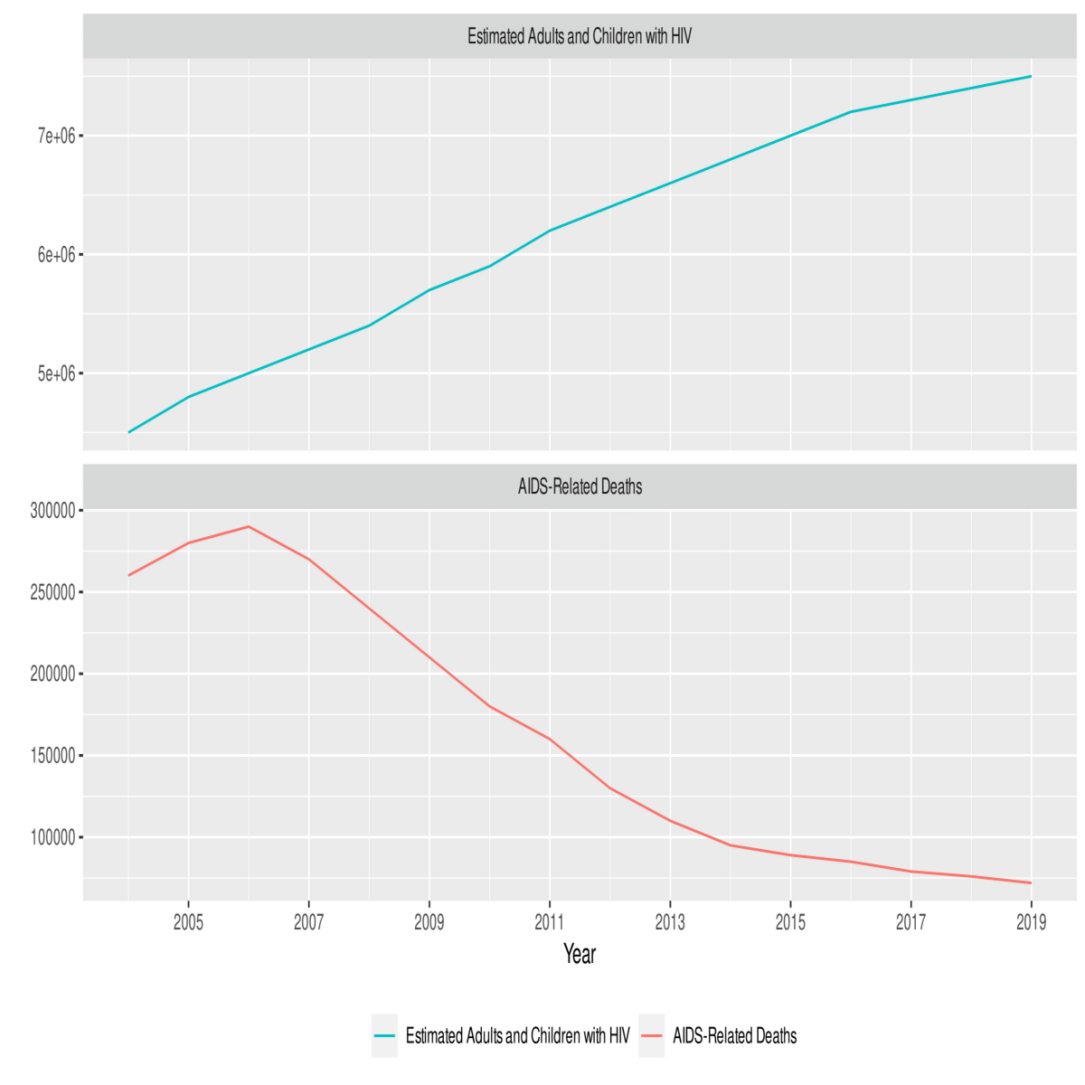
UNAIDS data on estimated adults and children living with HIV and AIDS-related deaths in South Africa [34]. UNAIDS: Joint United Nations Programme on HIV/AIDS.

Search term trends for “HIV” and “AIDS” from 2004-2019 in South Africa showed a decline in “AIDS” searches relative to “HIV” (*P*<.001; Figure 1). This observed online behavior mirrors progress on the ground for both HIV and AIDS (*P*<.001, association between “AIDS” searches and deaths), as there was a decrease in AIDS-related deaths (*P*<.001) and an increase in the number of people living with HIV (*P*<.001) in the country from 2004-2019 (Figure 2).

Some of the most important search queries (top and rising) searched for by South Africans on “HIV” and “AIDS” can be seen in Multimedia Appendix 1 and Multimedia Appendix 2, respectively. A complete visualization of the search data, regional interest in South Africa for the search terms “HIV” and “AIDS,” and rising and top search queries on “HIV” and “AIDS” can be accessed via Google Trends [30].

Similarly, on Wikipedia Afrikaans, for the years 2015-2020, there was greater interest in “HIV” than “AIDS” (Multimedia Appendix 3). 

## Discussion

### Principal Findings

This paper shows a statistically significant decline (*P*<.001) in search interest on Google for AIDS relative to HIV in South Africa. This trend mirrors progress on the ground in South Africa and is significantly associated (*P*<.001) with a decline in AIDS-related deaths and people living longer with HIV. This trend was also replicated on Wikipedia Afrikaans, where there was a greater interest in HIV than AIDS. The data on the search terms “HIV” and “AIDS” in South Africa (2004-2019) reveal a decrease in interest over time in AIDS-related search queries relative to HIV-related search queries. This mirrors progress on the ground in South Africa, where AIDS-related deaths have reduced over the years (2004-2019) and people are living longer with HIV due to life-changing medications. Similarly, behavioral interventions through messages delivered through media sources in South Africa, including the internet, have increased the number of people with an accurate understanding of the differences between HIV and AIDS [3]. In response to these behavioral interventions, a proportion of the populations in these countries will use the internet via search engines to seek information on HIV and AIDS. From the earliest data available on Wikipedia Afrikaans (2015-2020), there has been a greater interest in HIV than AIDS in South Africa.

### Comparison to Prior Work

This work contributes to the growing literature on infodemiology**,** which seeks to employ search engine data in the service of health information seeking, forecasting, and surveillance [35]. Previous research has covered a wide variety of infectious diseases such as influenza [36], dengue [37], norovirus [38], COVID-19 [39], and sexually transmitted infections [40], as well as other topics like cancer [22,23], dementia [18], and mental health [20]. Among the limited literature exploring HIV/AIDS with internet search data, the methodology is often restricted to qualitative [41] or parametric methodology [42], with limited research on Africa. Our work contributes to the limited literature (eg, [1,43]) on HIV/AIDS in the African context. Moreover, the nonparametric methodology we adopted requires minimal assumptions about the data and its distribution, and is thus robust and adaptive. Our work contributes to the niche of infodemiological study on HIV/AIDS in Africa and South Africa in particular while possessing the potential to extend to other countries/regions or geographical resolutions.

### Further Directions

An important follow-up to this study might be to understand how South Africans search for health information on other diseases and topics of public interest on search engines. In addition, motivated by our findings in this work, we may further investigate and develop a statistically principled and coherent framework to use Google search data for forecasting HIV/AIDS trends. In the meantime, a quantitative (survey) and qualitative (interviews) study of how South Africans access HIV/AIDS information online is planned, to provide insights for the research question by using traditional research methods. This study aims to provide data across South African provinces, disaggregated by age and gender. Additionally, broadening the scope of future studies could be achieved by studying other search engines, social media, and knowledge repositories, as research shows that, for example, Wikipedia is a good reflection of medical interests of the lay population [44], search engine queries are a good way of estimating outbreaks [45], and social media platforms are an important source of medical information today [46].

### Limitations

This paper reports on how, in general, interest in the search terms “HIV” and “AIDS” mirrors the increase in people living with HIV and the decline in AIDS cases in South Africa. Here, we report some limitations of the study.

First, there is an acknowledgement that the population of health information seekers on HIV/AIDS online may be quite different from the offline population. For instance, not everyone searching for information on HIV/AIDS may be connected online or use search engines, and the number of people connected to the internet changed over the years of the study (2004-2019). Hence, this paper is not an exact mapping of the online behavior of all the people searching for HIV/AIDS information in South Africa. This limitation is common in research using online big data methods such as search engine data, where the nature of the specific platform from which the data were obtained shapes the results obtained [25]. To mitigate this, a follow-up quantitative and qualitative study of the HIV/AIDS health information seeking behavior of South Africans using Google is planned. This would provide data through traditional (non–big data) research methods, following best practices from digital big data research, where the best results are often obtained when traditional data collection methods like surveys are combined with big data methods to inform research inquiry [25].

Second, we also acknowledge that the observed trends in the Google search data could be explained by the general decrease in people’s interest in such diseases and public health matters. To adjust for this, we used the search term “flu” as a control and the declining trend of the search term “AIDS” remained significant after adjustment (*P*<.001). In addition, this paper acknowledges the effects of media coverage on interest in specific terms, which is not accounted for in this paper.

Third, this paper focuses only on studying the association between the Google search interest in HIV/AIDS and cases/deaths. We acknowledge that causal relationships should not be drawn due to the limitations of the data. In addition, we acknowledge that although we used the Google Trends category “health, web search” to refine the search results for “AIDS,” the returned results may contain other meanings or associations of the word “AIDS,” as seen in some entries in Multimedia Appendix 2.

### Conclusions

A major obstacle to combating the impacts of disease in developing countries is the paucity of high-quality health data, particularly for understanding people’s health information needs [1]. People’s information needs and their everyday concerns are often expressed via search engine queries as millions go online to meet their health information needs. This paper shows a statistically significant decline (*P*<.001) in search interest on Google for AIDS relative to HIV in South Africa. This trend mirrors progress on the ground in South Africa and is significantly associated (*P*<.001) with a decline in AIDS-related deaths and people living longer with HIV. This trend was also replicated on Wikipedia Afrikaans, where there was a greater interest in HIV than AIDS.

In developing country contexts where high-quality data on the health information needs of people is often lacking, understanding the population’s use of search engines to meet health information needs can provide useful data. Investigating people’s use of search engines to meet information needs can also reveal if education and programming efforts have been effective. Consequently, a natural progression of this study might be a quantitative and qualitative study of the use of search engines and other online information sources to meet HIV- and AIDS-related health information needs in South Africa, which might mitigate platform-specific limitations of findings as outlined in the Limitations section.

## Appendix

Multimedia Appendix 1 Top and rising related queries for HIV, 2004-2019.

Multimedia Appendix 2 Top and rising related queries for AIDS, 2004-2019.

Multimedia Appendix 3 Wikipedia Afrikaans pageview statistics on HIV and AIDS 2015-2020 (accessed November 5, 2020).
