# Supplementary material for: Searching for HIV and AIDS Health Information in South Africa, 2004-2019: Analysis of Google and Wikipedia Search Trends
Source: JMIR Form Res. 2022 Mar 11;6(3):e29819. doi: 10.2196/29819 (PMC8956998; doi:10.2196/29819)
Supplement: Multimedia Appendix 1 [file formative_v6i3e29819_app1.docx]

**Multimedia Appendix 1. Top and rising related queries for HIV, 2004-2019**

| **Queries** |
| --- |
| hiv aids |
| Aids |
| Symptoms |
| hiv symptoms |
| hiv and aids |
| hiv positive |
| what is hiv |
| hiv test |
| symptoms of hiv |
| hiv cure |
| hiv in south africa |
| hiv treatment |
| hiv signs |
| hiv infection |
| hiv testing |
| early hiv symptoms |
| treatment for hiv |
| hiv medication |
| hiv test kit |
| hiv pills |
| hiv cure news |
| meaning of hiv |
| Cancer |
| impact of hiv and aids |
| hiv/aids |
| people with hiv |
| signs of hiv |
| hiv negative |
| what is hiv aids |
| what is aids |
| living with hiv |
| tb |
| hiv news |
| hiv aids in south africa |
| hiv meaning |
| hiv prevention |
| what causes hiv |
| people living with hiv |
| effects of hiv |
| how to get hiv |
| arv |
| hiv rash |
| hiv and pregnancy |
| hiv symptoms men |
| hiv positive news |
| hiv symptoms in men |
| sign of hiv |
| hiv symptoms women |
