# Supplementary material for: Searching for HIV and AIDS Health Information in South Africa, 2004-2019: Analysis of Google and Wikipedia Search Trends
Source: JMIR Form Res. 2022 Mar 11;6(3):e29819. doi: 10.2196/29819 (PMC8956998; doi:10.2196/29819)
Supplement: Multimedia Appendix 2 [file formative_v6i3e29819_app2.docx]

**Multimedia Appendix 2. Top and rising related queries for AIDS, 2004-2019.**

| **Queries** |
| --- |
| hiv |
| hiv aids |
| hiv and aids |
| aids in africa |
| aids in south africa |
| medical aids |
| what is aids |
| medical aids south africa |
| hiv symptoms |
| hiv aids symptoms |
| medical aid |
| hiv and aids in south africa |
| symptoms of aids |
| aids meaning |
| hiv cure |
| hiv and aids cure |
| visual aids |
| poverty |
| aids 2016 |
| meaning of aids |
| discovery medical aid |
| difference between hiv and aids |
| bonitas (health cover) |
| aids symptoms |
| what is hiv |
| what is hiv aids |
| hiv aids in south africa |
| aids day |
| hiv/aids |
| what is hiv and aids |
| aids cure |
| hearing aids |
| world aids day |
| people with aids |
| medical aids in south africa |
| impact of hiv and aids |
| cure for aids |
| aids foundation |
| symptoms of hiv and aids |
| teaching aids |
| hiv aids statistics |
| aids statistics south africa |
| causes of hiv and aids |
| aids stand for |
| definition of aids |
