# Supplementary material for: Searching for HIV and AIDS Health Information in South Africa, 2004-2019: Analysis of Google and Wikipedia Search Trends
Source: JMIR Form Res. 2022 Mar 11;6(3):e29819. doi: 10.2196/29819 (PMC8956998; doi:10.2196/29819)

**Multimedia Appendix 3. Wikipedia Afrikaans pageviews statistics on HIV and AIDS 2015 – 2020, accessed November 5 2020**.


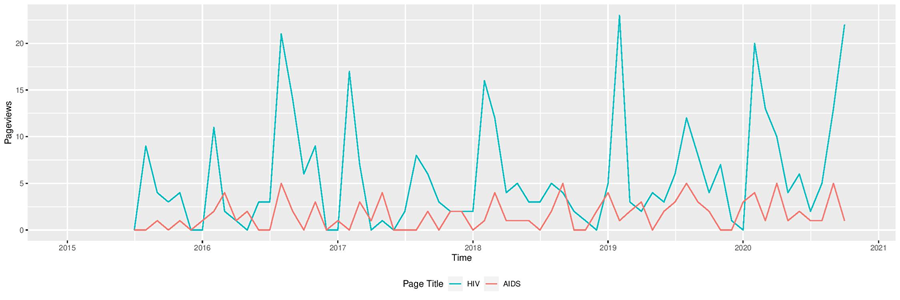

Supplement: Multimedia Appendix 3 [file formative_v6i3e29819_app3.docx]
